# User Preferences for Content, Features, and Style for an App to Reduce Harmful Drinking in Young Adults: Analysis of User Feedback in App Stores and Focus Group Interviews

**DOI:** 10.2196/mhealth.5242

**Published:** 2016-05-24

**Authors:** Joanna Milward, Zarnie Khadjesari, Stephanie Fincham-Campbell, Paolo Deluca, Rod Watson, Colin Drummond

**Affiliations:** ^1^ Addictions Department Institute of Psychiatry, Psychology and Neuroscience King's College London London United Kingdom; ^2^ Health Innovation Network London United Kingdom

**Keywords:** alcohol, drinking, young adults, mhealth, brief intervention, apps, smartphone, digital, focus group

## Abstract

**Background:**

Electronic screening and brief intervention (eSBI) is effective in reducing weekly alcohol consumption when delivered by a computer. Mobile phone apps demonstrate promise in delivering eSBI; however, few have been designed with an evidence-based and user-informed approach.

**Objective:**

This study aims to explore from a user perspective, preferences for content, appearance, and operational features to inform the design of a mobile phone app for reducing quantity and frequency of drinking in young adults engaged in harmful drinking (18-30 year olds).

**Methods:**

Phase 1 included a review of user reviews of available mobile phone apps that support a reduction in alcohol consumption. Apps were identified on iTunes and Google Play and were categorized into alcohol reduction support, entertainment, blood alcohol content measurement (BAC), or other. eSBI apps with ≥18 user reviews were subject to a content analysis, which coded praise, criticism, and recommendations for app content, functionality, and esthetics. Phase 2 included four focus groups with young adults drinking at harmful levels and residing in South London to explore their views on existing eSBI apps and preferences for future content, functionality, and appearance. Detailed thematic analysis of the data was undertaken.

**Results:**

In Phase 1, of the 1584 apps extracted, 201 were categorized as alcohol reduction, 154 as BAC calculators, 509 as entertainment, and 720 as other. We classified 32 apps as eSBI apps. Four apps had ≥18 user reviews: Change for Life Drinks Tracker, Drinksmeter, Drinkaware, and Alcohol Units Calculator. The highest proportion of content praises were for information and feedback provided in the apps (12/27, 44%), followed by praise for the monitoring features (5/27, 19%). Many (8/12, 67%) criticisms were for the drinking diary; all of these were related to difficulty entering drinks. Over half (18/32, 56%) of functionality criticisms were descriptions of software bugs, and over half of those (10/18, 56%) were for app crashing or freezing. Drinksmeter and Alcohol Units Calculator were the most highly praised apps overall (23/57 and 22/57; 39% of praise overall). In Phase 2, two main themes were identified. The meaningfulness theme reflected how young adults thought apps needed to be tailored to the interests and values of their age group, particularly emphasizing content and feedback around broader health and well-being factors such as exercise, diet, and image. The community theme suggested that young adults want to be able to engage with other app users, both in groups of friends and with online users for motivation and support.

**Conclusions:**

Targeted and relevant information and feedback, in addition to easy-to-use monitoring tools, were found to be important features of a mobile phone app to support a reduction in drinking. Future app development should consider tailoring all app aspects to the needs of young adults, considering broader well-being monitoring tools and online community functions.

## Introduction

The Internet has been found to be an effective vehicle for the delivery of screening and brief intervention (SBI) to reduce alcohol consumption. Meta-analyses demonstrate electronic SBI (eSBI) to be effective in reducing alcohol consumption by 1-2 drinks per week after 6 months compared to controls [1,2]. eSBI delivered via mobile phone, tablet, or computer can be delivered discreetly and flexibly to large numbers of the population in need at a competitive cost, without reliance on the time of health care staff, with whom there are well-recognized barriers to implementation [3,4]. Research suggests that eSBI is the preferred delivery medium for alcohol SBI in young adults [5].

The ubiquity of mobile phones provides a further vehicle for the delivery of eSBI via digital apps. It is estimated that at least 76% of young people aged 15-34 own a smartphone in the United Kingdom [6], with 77% of young people who own a smartphone using apps, the highest among all age categories [7]. However, nearly 90% of alcohol-related apps available to download encourage alcohol consumption, for example through drinking games [8], with few alcohol apps that use evidence-based behavior change techniques (BCTs) to target harmful alcohol use [9,10].

Developing effective and engaging eSBI apps is a new challenge for researchers. Few randomized controlled trials of alcohol apps, as opposed to Internet-delivered SBI, have been published, and those that have provide ambiguous evidence on their effectiveness to reduce alcohol consumption, with studies reporting reductions, increases, and no-change in alcohol consumption [11-13]. A major issue with app development is sufficiently engaging the target population with the app content and features: while app usage continues to rise [14], 95% of apps are not used more than a month after download [15].

For an app designed to support a reduction in drinking, it is important to look to the informatics field for guidance on developing apps. User-centered design (UCD) [16] is a systematic app development method that focuses on the relevance, needs, and preferences of the target user. UCD involves consideration of the user at every stage of the design process including iterative cycles of focus groups, prototyping, and user testing.

Previous research has focused on alcohol apps’ adherence to evidence-based guidelines or app effectiveness in reducing drinking [9-11]. To the authors’ knowledge, no research has explored the preferred content of alcohol apps from a user perspective. The aim of this study is to determine preferences for content, visual appearance, and operational features to inform the development of an eSBI app targeting harmful drinking young adults.

The specific objectives addressed by this paper were to (1) quantitatively identify which eSBI app content, operational features, and esthetics were highly rated, criticized, and in need of improvement, through a content analysis of user feedback, and (2) qualitatively identify the preferences for content, operational features, and esthetics of young adults who drink harmfully, through focus groups.

## Methods

### Phase 1

#### Review of User Reviews of Alcohol eSBI Apps

The Apple iOS (mobile operating system) and Google Play platform allow users to download apps, assign a star-rating, and provide review comments. User reviews provide an important source of feedback for app developers and prospective app purchasers, typically containing positive and negative feedback on content, functionality, and quality of the app [17,18]. These user reviews provide a rich source of information to support app development from a user perspective and are a key measure of the app’s success [19].

#### Search Strategy and Data Extraction

Alcohol-related apps were extracted from the UK versions of Google Play and iTunes in April and May 2015 with the following search terms: “alcohol,” “drink less,” and “reduce drinking.” Search terms were based on those used in previous research [9], as well as additional terms suggested by members of the wider alcohol research team in the Addictions Department at King’s College London. The first 200 apps for each search term were extracted [9], which were presented in rank order. App name, price, ranking in search results, and number of user reviews were extracted.

Apps were considered eligible for inclusion if they were categorized as an eSBI app and their aim were to help users monitor and cut down their alcohol use. The objective of the paper was to inform the development of an electronic brief intervention for harmful drinkers; therefore, apps targeting dependent drinkers were excluded.

In Stage 1, apps extracted from the initial search were categorized from the description of the app provided by the developer as targeting alcohol reduction support, blood alcohol content (BAC) calculators, entertainment (drinking games, cocktail recipes, bar and restaurant finders), or other (all apps that had no content relating to alcohol, eg, Candy Crush, non-English apps, and apps for educational purposes such as apps for mental health professionals).

In Stage 2, eSBI apps were extracted from the alcohol reduction support category. The aim was to broadly include apps that included SBI components such as alcohol monitoring, goal setting, and normative feedback [20]. Apps were excluded if they used non-evidence-based methods for alcohol monitoring/support/reduction such as hypnosis and magic spells; were targeted at dependent drinkers; were eBooks/magazines/quotes; targeted multisubstance abuse or drugs; targeted a specific group (such as pregnant women); or claimed to provide alcohol therapy or counseling.

In Stage 3, apps were excluded if they duplicated across the same platform (ie, appeared more than once in the initial search across Google Play). Duplicate apps across the two search platforms (eg, Google Play and iTunes) were not excluded as the user reviews are different for each platform and could be included in analyses. Apps with no user reviews were excluded.

#### Analysis of User Reviews

Each app was coded for content. A researcher downloaded the app onto an iPhone 6 and categorized each feature targeting alcohol monitoring and reduction support. Content analysis was used to code the app review content in order to create a quantitative description of the text [21]. The coding scheme for the user feedback review was adapted from themes identified in a previous review of app user feedback, developed by Pagano and Maalej [22], and through pilot coding of a random sample of 42 app user reviews. If any new codes emerged from the data, these were incorporated into the coding scheme. Each user review was then independently coded by 2 reviewers (JM and SFC) using the deductive coding scheme. The 2 coders had an 81% agreement rate across the coding categories. All discrepancies were discussed until 100% agreement was reached.

### Phase 2

#### Focus Groups

##### Design and Setting

Focus groups were chosen as the most appropriate method of data collection as they allow for a multiplicity of views to be shared, developed, and discussed, as well as allowing for consensus on a topic to be explored, which is not possible with a one-to-one interview qualitative design. Four focus groups were conducted at the Denmark Hill Campus of King’s College London. Ethical approval was obtained from the University Ethics Committee (ref. number HR14/150453).

##### Facilitators

Two members of the research team (JM, ZK, and RW) facilitated each focus group. The facilitators all have a background in delivering SBI and experience with developing electronic health interventions. All facilitators were experienced in conducting focus groups.

##### Participants

Young adults, aged 18-30 years who lived in South London and scored 16+ on the alcohol use disorders identification test (AUDIT) [23], a validated measure of alcohol consumption and related harm, were eligible to participate in the study.

##### Recruitment

Participants were recruited via paid online advertisements through Facebook (an online social networking website) and Gumtree (a free online classified advertising and social community website). The advertisements invited potential participants to take part in a focus group that would review different mobile phone apps available to help young adults reduce their alcohol use and examine how they could be improved. A link was provided on the advertisement that accessed an online prescreening survey. Potentially eligible participants completed the AUDIT as well as providing information on age, contact details, and address.

All potential participants who met the inclusion criteria, that is, aged 18-30, living in South London, and drinking at a level considered harmful (16+ on the AUDIT), were invited to participate in a focus group. Participants received £30 in High Street vouchers as compensation for their time. Travel expenses were recompensed.

One week before the focus group, participants were asked to download a specific eSBI app (selected from those with the most user reviews) and use it over the course of a week, thinking about what they liked and disliked about the app.

#### Data Collection

Written informed consent was obtained before commencement of the group. The facilitators introduced the project, outlined the aims, and highlighted the ground rules of confidentiality and mutual respect. Each focus group lasted for approximately 1.5 hours.

Semistructured topic guides were used to frame the group discussion. The first half of the focus group explored what participants liked and disliked about the app they had reviewed. This discussion was broadly organized by the topics of content, functionality, esthetics, information on drinking, and how the app could be improved. The second half of the focus group discussed the type of features the participants might include if they were designing their own eSBI app to help young adults reduce their alcohol use.

#### Data Analysis

Focus groups were recorded and transcribed verbatim by a professional transcription company. All data were coded using NVivo qualitative data analysis software (QSR International Pty Ltd. Version 10, 2012). A detailed thematic analysis was undertaken [24]. Both an inductive and deductive approach were used, incorporating the major themes (codes) from the app review as well as any additional themes emerging from the data. The transcripts were read through several times and coded deductively using the coding framework from the app review as well inductively for new themes. The codes were then systematically organized into broader themes and subthemes and re-examined by going back and forth between the data and the coding framework.

## Results

### Phase 1: Review of User Reviews of Alcohol eSBI Apps

#### Description of Apps

**Figure 1:**
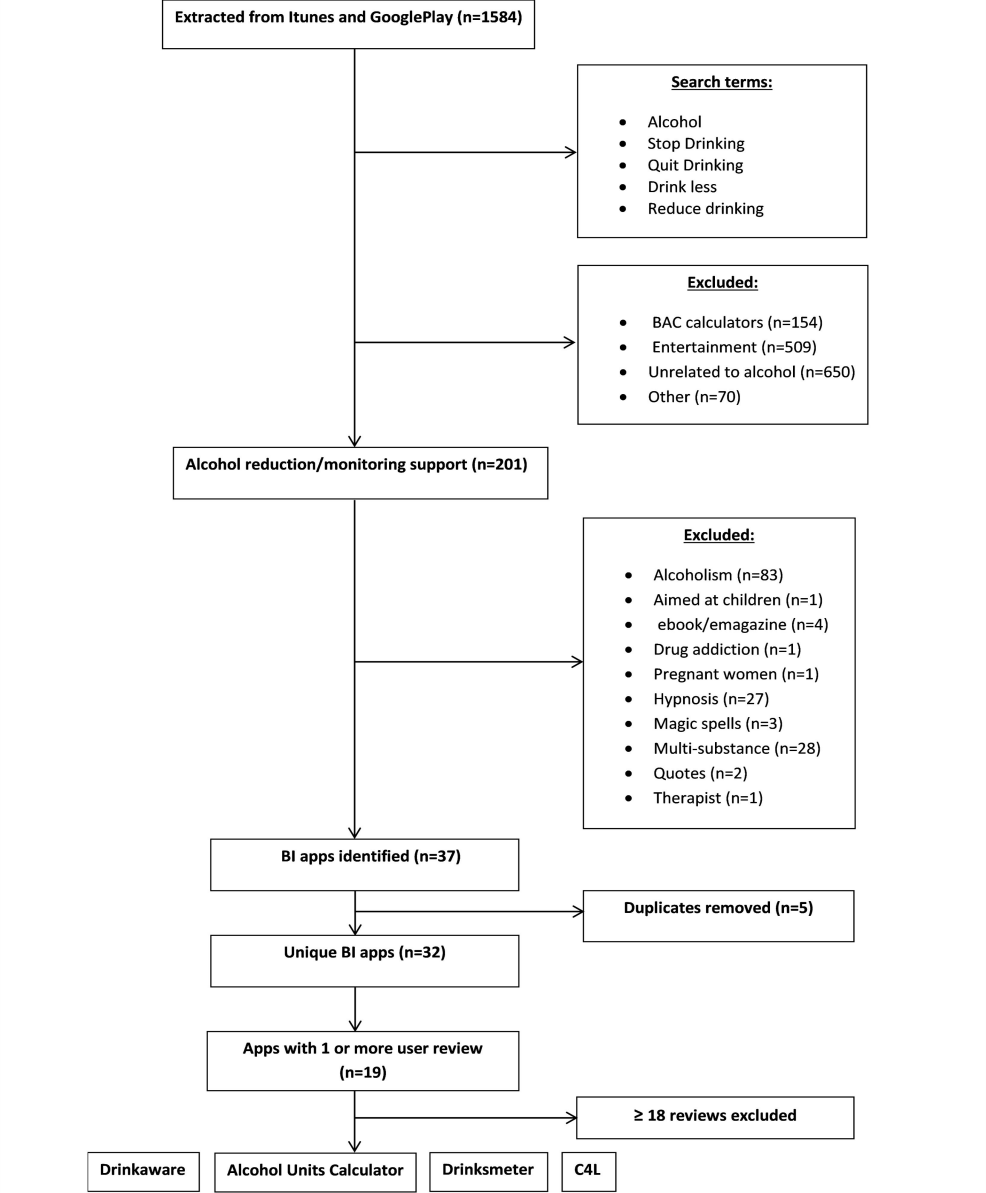
Flow diagram of apps selected for coding.

Of the 1584 apps extracted in the search, 154 were categorized as BAC calculators, 509 as entertainment apps, and 720 as other (see Figure 1). We categorized 201 apps as targeting alcohol monitoring and reduction. After excluding multisubstance-use apps, non-SBI apps, and duplicates across platforms, 37 apps remained (32 unique apps when excluding duplicates between platforms). Of the unique apps, 69% (22/32) were free apps. Nineteen of the apps had one or more user reviews. The majority of apps had low numbers of reviews (range 1-114, mean 18.0 reviews, SD 31.24); 74% (14/19) had 1-10 reviews, 5% 11-17 (1/19) and 21% had ≥18 (4/19). As the user reviews were typically brief, consisting of a few sentences of feedback, apps were included that had more than or equal to the mean number of reviews (mean 18) to provide sufficient data to conduct a content analysis.

Four unique apps were identified for final analysis: Drinkaware (114 reviews); NHS Change for Life (C4L) Drinks Tracker (95 reviews); Drinksmeter (21 reviews), and Alcohol Units Calculator (AUC, 18 reviews). The 18 most recent reviews were included, resulting in a total of 72 individual app user reviews for analysis. Drinksmeter had the highest user-rating out of five stars overall (mean 4, SD 0.3), followed by AUC (mean 3.6, SD 1.5), Drinkaware (mean 3.1, SD 1.30), and C4L with the lowest (mean 1.7, SD 1.1).

The apps varied in complexity and sophistication of content (see Table 1); however, all included at minimum a daily drinks tracker with graphical feedback on units (except Drinksmeter, which requested drinking level over the previous week only). Excluding AUC, all apps provided information on drinking risk level and recommended limits. C4L and Drinksmeter provided tips and advice for cutting down. Drinkaware and Drinksmeter provided feedback on calories, costs, and equivalents in unhealthy food. Drinksmeter provided normative feedback on weekly alcohol consumption, included a risk adjuster for mental health, medication, and drug use as well as administering the AUDIT and providing feedback. Drinkaware had additional features including the option to set goals, create “weak spots” via GPS data on the phone and information on alcohol support services.

**Table 1:** Description of app features.

Feature included	Drinkaware	Drinksmeter	AUC	C4L
Drinks tracker (daily)	Yes	No	Yes	Yes
Drinks tracker (previous week)	Yes	Yes	Yes	Yes
Information on drinking risks	Yes	Yes	No	Yes
Information on guidelines	Yes	Yes	No	Yes
Information on support services	Yes	No	No	No
Advice for cutting down	No	Yes	No	Yes
Normative feedback	No	Yes	No	No
Feedback: calories, costs, food	Yes	Yes	No	No
Risk adjuster	No	Yes	No	No
AUDIT	No	Yes	No	No
Goal-setting	Yes	No	No	No
Weak spots	Yes	No	No	No

#### Categories

A content analysis was performed based on the five primary coding categories defined in Table 2. All primary codes were subsequently coded into subcategories and then coded as either praise, criticism, or recommendation statements. A total of 194 meaning units were identified. Key findings are reported here (see Table 3 for a full breakdown of coding).

**Table 2:** Description of coding categories.

Category	Categorized references, n	Definition	Examples
Content	60	All text relating to content and features of the app such as the drinks diary, graph, and information provided.	“Only improvement would be to have the line graph over 28 days instead of just 7 days.”
Functionality	43	All text relating to operational features of the app such as saving, entering, and loading data. Descriptions of software bugs.	“Doesn’t work properly. Can’t get beyond the daily input page to see totals or goals etc”
Esthetics	7	All text relating to the visual appearance of the app.	“Plus points—Jazzy colour scheme—arguably prettier than the earlier more functional design”
General comments	24	All text relating either praise or criticism of the app that was non-specific.	“Great app”
Other	60	All content not relating to a topic above, eg, descriptions of app and usage patterns, who the app would benefit, questions to other users and developers, jokes, noise.	“Am a GP. Use this with my patients.”

#### Content Category: What Users Liked (Praise)

Nearly half (27/60, 45%) of references in the content category were praise for the apps. The highest proportion (12/27, 44%) was praise for the information and feedback provided in the apps, followed by praise for the drinks diary features (5/27, 19%).

Drinksmeter had the highest proportion of praise references for the information and feedback it provided (10/12, 83% of praise references). AUC was the most praised for its monitoring features (10/12, 83% of praise references for monitoring). Drinksmeter and AUC had the highest proportion of praise references for content overall, both with 41% (11/27) of praise references. C4L was the least praised in terms of content with 4% (1/27) of content praise references.

#### Content Category: What Users Disliked (Criticisms)

One-fifth of all content references were criticisms (12/60, 20%). The majority (8/12, 67%) were for the drinking diary. All of these criticisms were related to difficulty entering drinks, such as apps not providing enough brands or drink choices to enter consumption level, limited drink size choices, limited options to enter alcohol strength (Alcohol by Volume [ABV]), and not being able to enter more than one drink at a time.

Drinkaware received the highest proportion of content criticisms overall with 50% (6/12) of references. C4L received 42% (5/12) of content criticisms and AUC 8% (1/12). Drinksmeter did not receive any criticisms for content.

#### Content Category: Recommendations for Improvement

Thirty-five percent (21/60) of content references were recommendations. 62% (13/21) were recommendations for improvement of the drinking diary, followed by 24% (5/21) for the graph function (which tracks alcohol use over previous week/month). The most frequently cited recommendations were for functions to enter specific ABV values (4/21, 19%), followed by being able to store favorite drinks (3/21, 14%).

**Table 3:** Coding frequencies by category.

Category		Praise frequency, n (%)	Criticism frequency, n (%)	Recommendation frequency, n (%)
**Content (overall)**				
	Goal setting	0 (0)	0 (0)	1 (5)
	Information	12 (44)	3 (25)	2 (10)
	Monitoring-drinks diary	5 (18)	8 (67)	13 (62)
	Monitoring- graph	3 (11)	0 (0)	5 (24)
	Monitoring-general (praise/criticism)	4 (15)	0 (0)	0 (0)
	Positive reinforcement	0 (0)	1 (8)	0 (0)
	Reminders	1 (4)	0 (0)	0 (0)
	Rewards	1 (4)	0 (0)	0 (0)
	Tone	1 (4)	0 (0)	0 (0)
	General praise/criticism	0 (0)	0 (0)	0 (0)
Total		27 (100)	12 (100)	21 (101)
**Functionality (overall)**				
	Data-entering	0 (0)	1 (3)	0 (0)
	Data-losing	0 (0)	4 (13)	0 (0)
	Data-saving	0 (0)	3 (9)	0 (0)
	Data-importing or exporting	0 (0)	0 (0)	2 (66)
	Description of bug	0 (0)	18 (56)	0 (0)
	Processing speed	0 (0)	3 (9)	1 (33)
	General praise/criticism	8 (100)	3 (9)	0 (0)
Total		8(100)	32 (99)	3 (99)
**Esthetics (overall)**				
	Color	2 (50)	0 (0)	0 (0)
	Text	0 (0)	1 (33)	0 (0)
	General praise/criticism	2 (50)	2 (66)	0 (0)
Total		4 (100)	3 (99)	0 (0)
General comments		18(100)	6 (100)	0 (0)

#### Functionality Category: What Users Liked (Praise) and Disliked (Criticisms)

No users praised specific components of the functionality of the apps. Eight unique references (100% of functionality praise references) praised general functionality components. Due to the small sample size, this was not examined between apps.

The majority of criticism references (18/32, 56%) were related to descriptions of bugs in the software. The most commonly criticized bugs were issues relating to the app crashing or freezing (10/18, 56%).

#### Esthetics Category

Relatively few users reported on esthetics across the four apps. There were 7 references in total; these were mostly split over general praise comments (29%) and general criticisms (29%).

#### Most Liked and Disliked Apps

All of the praise and criticism references across the three categories (content, functionality, and esthetics) were aggregated.

There were 57 praise references overall (43% of all references in total). Drinksmeter and AUC received the most praise with 40% (23/57) and 39% (22/57) respectively, followed by Drinkaware with 14% (8/57).

There were 53 criticisms overall (40% of all references in total). Both Drinkaware and C4L received considerably more criticism (24/53, 45%, and 21/53, 39%, respectively) than Drinksmeter and AUC (2/57, 4% and 6/57, 11%) respectively.

### Phase 2: Focus Groups With Young Adults Engaged in Harmful Drinking

#### Recruitment

A total of 200 people completed the online screening survey; 117 from an advertisement placed on Gumtree, 83 via Facebook. In total, £80 was spent on Gumtree recruitment and £100 on Facebook recruitment. The advertisement invited anyone who drank alcohol to participate in a focus group about mobile phone apps for drinking reduction. Nearly three-quarters (146/200, 73.0%) were female. Over half (105/200, 52.5%) were employed (full-time or part-time), 22 were unemployed (11%), and 73 were students (37%). Of these, 81 (40%) had a self-reported score of 16 or more on the AUDIT, were between 18-30, and lived in South London. In total, 36 eligible participants signed up to one of the four focus groups.

#### Participant Characteristics

Twenty-one participants attended one of four focus groups over a 1-month period in June-July 2015. Of the 21 participants, 18 (86%) were female, 12 (57%) were employed, 7 (33%) were students, and 2 (10%) were unemployed. The mean AUDIT score across the participants was 20 (SD 5.0).

#### App Selection

The apps were selected based on the four apps identified in the user app review (Drinkaware, Drinksmeter, C4L, and AUC). Only free apps were included in the analyses. Drinkaware was randomly selected to be reviewed twice. C4L was reviewed by 4 participants, Drinkaware by 10 participants, and Drinksmeter by 7 participants.

#### Young Adults’ Views on the Development of an ESBI App to Target Harmful Drinking

Two main themes emerged from the data: the theme of meaningfulness and the theme of community. Key findings are reported below. See Table 4 for a detailed breakdown of themes.

##### Meaningfulness

The meaningfulness theme broadly describes the opinion of the participants that an approach that tailors all content and features to the target user is inherent to any successful eSBI app. The “meaningfulness” theme is divided into three further subthemes: information and feedback, goals, and monitoring.

###### Information and Feedback

The majority of participants felt that the information and feedback provided to young adults about drinking was often not meaningful. For example, government drinking guidelines (eg, recommended unit consumption) and information on risk categories (such as “high risk”) were felt to be unrealistic and irrelevant to the participants:

Well also the levels…I know it’s the government levels and the medical stuff but like nobody sticks to them. Well obviously some people do. So, you know, you put in a couple of drinks for two nights and you’re already increasing or higher risk or something, which is unrealistic. A normal night for normal people is high risk, well that’s not going to help me, I don’t think, because that’s standard. So I think just to monitor your drinking and stuff, fine, but I think if you actually want to use it to cut back, just saying you’re at higher risk, well that’s meaningless because everybody I know is.

**Table 4:** Description of main themes and subthemes from focus groups.

Themes	Description	Subthemes 1	Description	Subthemes 2	Description
Meaningfulness	Apps need to be designed as meaningful and tailored to young adults.	Risk categories and government recommendations	Young people felt that government guidelines are unrealistic in light of UK drinking cultures.	n/a	n/a
		Information and feedback	Must provide information and feedback which is interesting to young people.	Wellness and lifestyle	Young adults are interested in factors such as weight gain; exercise; calorie and sugar content; impact upon image; junk food and exercise equivalents; costs; physical, psychological, and social impact; positive information; safety tips and sober things to do.
		Information and feedback	See above.	Personalization and tailoring	Information and feedback needs to be tailored to users’ demographics. Users would like to be able to set up own information preferences.
		Information and feedback	See above.	Presentation	Information needs to be succinctly displayed, not wordy, bullet-pointed, easy to read. Users would like the option of information pop-ups.
		Goals	Goals should be meaningful to young people, focus on short-term and long-term health risks and include lifestyle and wellness goals.	Personalization and tailoring	Set own goals and write personalized messages to yourself for motivation. Personalize timings and frequency.
		Goals	See above.	Prompts for goals	Option to set up reminders for goals at important times, eg when on a night out.
		Monitoring	Monitoring features should include a drinks diary and graph.	Drinks Diary	Data should be easy to enter including prompts and reminders; more choices of brands and drinks; barcode scanner.
		Monitoring	See above.	Graphs	Plot other health/well-being information that is relevant to drinking, eg mood; costs, and events.
		Esthetics	Young people wanted an app that was stylish and well-designed, with options to personalize and tailor the look of the app. They also requested the option to set up a user profile.	n/a	n/a
Community	Creating an online community of young people who want to reduce their drinking.	Support and motivation	The most successful way to cut down drinking is with the support of other people. This should be integrated into apps for young people.	n/a	n/a
		Group goals	Feature that allows an individual user to join an online group and work towards a specific goal, eg, “spend less money on alcohol” or “have a drink-free weekend”.	Friends	Users should be able to set goals with groups of friends.
		Group goals	See above.	Online users	Users should have the option to join online groups with a dedicated goal via a goals “forum”.
		Autonomy and privacy	Group goals should be opt-in and users can choose to set up private goals only.	n/a	n/a

One reason for the information not being meaningful to the participants was that because episodic binge-drinking among young people is perceived as such a socially accepted and entrenched activity, the drinking guidelines are thought to be unachievable and consequently not relevant to young people.

Another frequently cited reason was that the information and feedback on risks provided by the apps was generic and not tailored to specific target groups. Participants discussed how important it was for information and feedback to be tailored to them as individuals, making it more relevant to them:

I don’t know how to put it. It’s not that I disagree with it, it’s just that I understand that it’s taken from data over like millions of people potentially and that an individual is very different to a million people, and some people have a great capacity for drinking huge amounts and being absolutely fine.

On the other hand, apps that the participants praised the most for information and feedback (such as Drinksmeter) provided feedback on broader lifestyle and well-being factors such as exercise, alcohol-related weight gain, and the sugar content of drinks:

Having something other than the units like calories or sugar that you’re interested in, because ‘unit’ isn’t really a big factor for some people, like for me it’s not something I would count or particularly are worried about, so to use it I would need another factor.

This was a common theme throughout the focus groups. While participants were aware that heavy drinking put them at a higher risk of a variety of health problems, they also explained how, as young people, it was difficult to relate to such long-term health warnings. The shorter-term effects of alcohol on factors such as lifestyle and well-being, which young people value, would provide more motivation to cut down. Indeed, the effects of alcohol on participants’ physical looks were a key factor in making young people think about reducing their drinking:

It would say what I’m going to look like in five years, what I’m going to look like in ten years, because people are a bit vain as well, if you play into that. If it can show me if I drink at this level what I’m going to look like because of it in five or ten years that would probably make me cut down. That would probably make me be a bit healthier.

While participants did think it necessary to provide information and feedback on the negative consequences of drinking, in particular providing more “shocking” information similar to smoking health campaigns, the participants also reported wanting positive information on the benefits of not drinking

Yes, so giving people positive reasons not to drink and then negatives; like negative reasons would be: it’s bad for your health; you spend too much money. Positive things would be: you can achieve more in sports in fitness; you feel more confident.

Again, this links into the concept of wellness, in that reducing drinking is a broader lifestyle change undertaken to improve the quality of a person’s life in a variety of ways, from being able to exercise more, be more efficient at work, have fewer hangovers and improve their mental and physical health.

###### Goals

For the participants, being able to set personalized goals that were meaningful to them as individuals was paramount to successful alcohol reduction. Often participants would report that setting goals purely to reduce the number of units of alcohol consumed per week was not enough motivation for them to cut down.

I think then it’s making it a bit more than just a drinking app; there’s not that much incentive to cut down on drinking but when you make it more about your whole lifestyle then you are more likely to use it.

All participants suggested that users need to be able to set their own goals and reminders and be able to write themselves personalized messages for motivation. This included goals around other issues related to alcohol such as calories and costs:

It’s about creating something that people are actually going to use, not just bored with it or annoyed with [it], it’s something that’s got to be relevant. Like being able to set your own goals and potentially put your own messages on it…Being able to put a message into this app to remind me, "Just don’t have a drink tonight because you’re doing this tomorrow and you’ve already spent ‘x’ amount this week might be quite good."

Once again, goals linked into the concept of wellness and lifestyle, making alcohol reduction a channel through which to improve overall quality of life. For it to be meaningful to the participants, they must be able to tailor their goals to their priorities of what they want to achieve.

###### Monitoring

Consistently reported by participants was that all of the apps made it difficult to enter drinks into the drinks diary. This was typically because the apps were not designed around the drinking habits of young people. For example one female participant, talking about the C4L app, reported that it did not include the types of drinks that she consumed:

I was inputting that I had had some cider and I couldn’t remember what strength it was, I knew it was a strong cider and I don’t know if I’ve used the same app as you guys, ‘My Fitness Pal’ but I had a diet and exercise app that had like actual brand names and sizes, that would have been really useful so that I could have just chosen Henry J Weston then I would have known it was this brand so it was this percentage, because I think I definitely probably underestimated what it was.

Nearly all of the participants mentioned that the apps did not provide reminders to enter drinks. As the majority of participants’ drinking was done when out in pubs/bars/ clubs with friends, they reported that they simply forgot to enter the drinks throughout the night:

I used it every day but there wasn’t any prompt or anything, so I tended to forget and then put it in the next day.

Providing a well-designed graph to monitor alcohol consumption was also important to participants. It was suggested that the graph should provide additional information on well-being factors such as costs, mood, and the option to input important events such as birthdays to get a better understanding of their drinking:

Even if you put info you can kind of relate to why you drink more; sometimes like this money, you know, you just broke up with your boyfriend so it’s really high and, you know, things like that. Or you’ve had loads of birthdays in that month so you keep going out, or you’re celebrating finishing uni. Oh yeah, there could be a graph for financial...Yeah, that would scare me.

Overall, what is demonstrated in the meaningfulness theme is that young adults feel that the current eSBI apps available to download do not provide the type of information or functionality of features, which are pertinent and targeted to the needs of young people.

##### Community

The second major theme was around the idea that to build a successful app, it must engage with the wider community of young people trying to cut down their drinking. Participants felt that in order to have the highest chance of success of reducing their drinking, it is paramount to have the support of other people around them:

I use a running app and it’s a similar thing… when I run a race there was a lot of people that were in the group and we all kind of supported each other, so it’s a similar thing and it motivates you to keep using it because you’ve got people on there that like what you do.

As reflected in the meaningfulness theme discussed above, young people expressed the opinion that the most relevant aspects to them when considering cutting down on alcohol are broader lifestyle factors; young people want to be able to share these personal values via social media communities:

It’s about making you feel better, rather than your alcohol use…that’s where you link it into fitness and health and lifestyle because there’s a massive Internet forum for that; people want to shout about that so I think if you link it into that more people will actually go on it and check what other people have done that is healthy today, rather than what have people been drinking today.

In addition, participants wanted a personalized program of community support, which they could develop and manage themselves. This ranged from being able to set up goal-based groups with friends, to joining online groups that had similar goals to them:

You have everyone on there in the one community, everyone who joins is automatically in it, and you can talk to everyone who you want and your friends can join in, and if you want to create a group you can or if you want to add a group you can do what you do on Facebook and like send a message or send a request.

However, it was equally important for participants to be able to manage this autonomously, having the option of creating individual goals, as well as joining groups of their choosing:

It depends how the individual uses it. Like if they want to join all these groups, then they can, and they can be a member of loads of things and talk to loads of people but if they don’t want to do that and they just want their own personal profile and their own personal goals and just go on there to check how they’re doing. Then they can do that so they don’t feel pressured to spend hours on there.

The participants made it clear that they wanted to be in control of the app, and this was reflected across the different themes. There were also strong opinions that anonymity and privacy relating to online safety were essential, in that users wanted to be able to control which personal information was made public. Being able to personalize and tailor every aspect, from choosing the type of information provided to them, setting up their own user profiles to selecting both their goals and the way they achieved them, whether alone or in a group were integral to a successful app.

## Discussion

### Principal Findings

The aim of this paper was to determine preferences for content, functionality, and visual appearance for an eSBI app to help harmful drinking young adults reduce their alcohol use. Phase 1 conducted a review of user feedback reviews on the iTunes App and Google Play stores, examining what users praised and criticized about eSBI apps, and recommendations for improvement. Phase 2 conducted a series of focus groups with young adults engaged in harmful drinking, exploring their views on current eSBI apps, and determined optimal features, appearance, and functionality to support them to reduce their drinking.

### User App Review

The review generated three main findings. First, the element of eSBI apps that users praised most highly and most frequently was information and feedback. This suggests that good quality information and feedback is highly valued and desired by app users. Drinksmeter, which provides information and feedback on calories, costs, and their equivalents in unhealthy foods as well as normative feedback on alcohol consumption and a risk adjuster for mental health, received the highest number of this praise. This finding is consistent with the focus groups where Drinksmeter was also highly praised for its information and feedback. Future apps should pay close attention to the quality and type of information and feedback provided.

The second key finding was that users wanted a well-designed and easy-to-use drinks diary; all of the criticism for the drinks diary was for difficulty entering drinks, a finding that was also reflected in the focus groups. Monitoring of alcohol use is an important BCT to support reduction and is associated with greater effect sizes in SBI [20]. Future apps should include a range of data input options that allow users to enter drinks in the way that is easiest for them. Examples would be for users to be able to store their favorite drinks, be able to enter precise values for ABV and quantity, and have a broader selection of brands and types of drinks.

The third main finding was that what users disliked the most in terms of functionality was apps with software bugs. The most commonly criticized bug was for apps that crashed and froze. This is consistent with previous research; a study of over 250,000 reviews from the 20 most popular apps on the AppStore reported that functionality issues are the most criticized feature of an app, with app crashing being ranked number 3 of 12 [17]. An app that crashes often is likely to be deleted by the user before any engagement with the core objective, such as alcohol reduction, has occurred. App developers need to carefully test and improve the functionality of apps before releasing them for download or risk losing potential users in need.

Weaver et al [8] identified 44 alcohol reduction apps (in April 2012) and Crane et al [9] identified 91 alcohol reduction apps (in May 2014). Our study identified 201 apps (before exclusions) targeting alcohol monitoring and reduction. This would suggest that the alcohol app market has risen by 350% in 3 years. The current review did not examine adherence of apps to evidence-based guidelines, but as previous research reports, a large proportion of these apps are not evidence-based [8,9]. Apps endorsed and designed by health care and academic institutions are greatly needed.

### Focus Groups

Analysis of the focus group data identified two main themes to consider when developing an app for young adults engaged in harmful drinking. First, all components of the app should be developed with the user in mind, so that the app is meaningful to the target group. In this study, young adults reviewed four apps that were not tailored to specific groups. The young adults unanimously agreed that this approach alienated them as younger users. They felt that the information provided was often meaningless as it did not take into account individual-level factors such as demographic characteristics, mental and physical health, as well as the drinking habits of young adults. Generic risk categories, advice, and recommendations for units were intangible to the participants, as they felt they did not consider in a realistic way how young adults drink. This finding is confirmed by another recently published qualitative study reporting that drinking guidelines lacked relevance and were felt to be unrealistic by adult drinkers [25]. In order to capture the attention of young adults, the risks and recommendations about drinking need to be presented in a manner that is relevant and meaningful to young adults; otherwise, such information is ignored. Future research needs to explore what these messages might look like.

On the other hand, what young adults wanted was information, goals, and guidance related to issues that were important to them. Participants’ valued the positive benefits of not drinking such as maintaining one’s physical appearance, being able to exercise more, saving money, not putting on weight, and limiting bad hangovers. These broader lifestyle and well-being factors, targeted at short-term health and image improvement, were most highly rated as providing motivation for drinking reduction. This was reflected in both the types of information and feedback young adults preferred, and the types of drinking goals they wanted to set for themselves. While some eSBI programs do touch on these factors, such as providing calorie feedback, the current study shows how, for young adults, it needs to be at the core of the intervention for maximum impact.

This research is consistent with young adults’ changing attitudes towards their health. A recent report suggests that one of the reasons for reductions in drinking over the last 10 years in 16-24 year olds in England is because young people have a greater awareness of the negative risks associated with drinking alcohol [26]. Equally, 60% of adults self-track their health data; such as weight, diet, or exercise routine [27], highlighting the importance of leading a technology-supported healthy lifestyle to the modern adult. These values were reflected in our study, suggesting that such lifestyle factors and their link to alcohol should be included in future eSBI apps.

The second major theme was that young adults wanted to be able to draw on the support of others to help them drink less. This was in relation to being able to give encouragement and motivation to other users, for example, through a feature similar to the Newsfeed on Facebook, as well as having the option to set up and join groups with their friends and the wider online community to achieve joint goals. This finding is consistent with previous research from other health care sectors. Both a study that designed a sexual health website [28] and another that designed an app for diabetes self-management in adolescents [29] reported how the target group wanted to be able to interact with and provide support to other users online.

Having a community support feature is a recognized BCT [30] that, when delivered via an app, is a component traditional face-to-face BI does not provide. Thus, it has the potential to improve the quality of the SBI intervention. Furthermore, wanting to have an app that has the capacity to engage with the larger network of online users is consistent with the popularity of social media apps, particularly among young adults [31]. Connecting online with other users is part of the fabric of day-to-day app and Web usage for young adults, and it is reasonable that any new apps should be designed with the target group’s technology usage patterns in mind.

### Limitations

This study has a number of limitations. There were surprisingly low numbers of reviews available for alcohol eSBI apps suggesting that generalizations beyond the current study should be taken with caution. As this is the only review of its kind in the alcohol field, the authors believe that the data are important, particularly as the results are confirmed by both previous research and the focus group data, and can be enhanced upon with future research. Furthermore, it was not possible to limit the user feedback reviews to young adults; therefore, the opinions expressed are not specifically targeted to this age group. This limitation was overcome by targeting Phase 2 only at young adults. Only the first 18 reviews for each app were selected to ensure the same number of reviews were coded across the four apps. Therefore, reviews of apps with more than 18 reviews were excluded. While this may have affected the direction of the results, the authors suggest that the reviews coded represent the most current version of the app, and therefore the most up-to-date reviews. Earlier reviews may have provided feedback on obsolete issues and content. It is also noted that as the app market is ever expanding and transforming, the apps reviewed represent a snapshot in time, and the opinions expressed may have already been subject to change.

Despite attempts to recruit a demographically representative sample, the focus groups included more female participants. This may have affected the direction of discussion and the development of key themes. The AUC app (a paid app) was excluded. This limited the range of apps reviewed, which may have excluded important feedback from the results. Approximately half of all participants who applied to join the study had a self-reported score of 16 or more on the AUDIT. This is higher than the prevalence rates of harmful drinking reported in previous research [32]. Participants potentially inflated their alcohol use to be able to enter the study, suggesting that the AUDIT scores are not representative of the sample’s true drinking level. Conversely, due to self -selection bias, the sample may indeed have had higher drinking levels than the general population and therefore the preferences for content may reflect only the opinions of harmful drinkers with higher AUDIT scores. Additionally, only four focus groups were conducted with a total of 21 participants; however, the authors agreed that saturation was reached with the data and that the findings are transferable to other groups of young adults, particularly those living in London, who are studying or employed full or part-time. As a limitation to the study overall, aspects of esthetics of the apps were not discussed in detail, due to the nature of the topics that arose, and it is hard to draw any firm conclusions from the data on this aspect.

### Conclusions

This paper has provided a unique contribution to the field of eSBI by determining, from a user perspective, preferences of young adults for app content and functionality. Good-quality, relevant, and targeted information is paramount, as are easy-to-use features and options to engage with the wider online community. It is hoped that this research will inform the development of future mHealth apps and increase the availability of evidence-based mHealth products on the market.

## Abbreviations

ABV: alcohol by volume
AUC: alcohol units calculator
AUDIT: Alcohol Use Disorder Identification Test
BAC: blood alcohol content
BCT: behavior change technique
C4L: Change for Life
eSBI: electronic screening and brief intervention
SBI: screening and brief intervention
UCD: user-centered design
